# Local level inequalities in the use of hospital-based maternal delivery in rural South Africa

**DOI:** 10.1186/s12992-014-0060-1

**Published:** 2014-07-15

**Authors:** Sheetal Prakash Silal, Loveday Penn-Kekana, Till Bärnighausen, Helen Schneider

**Affiliations:** 1 Department of Statistical Sciences, University of Cape Town, Cape Town, South Africa; 2 Centre for Health Policy, School of Public Health, University of the Witwatersrand, Johannesburg, South Africa; 3 Department of Global Health and Population, Harvard School of Public Health, Boston, USA; 4 Wellcome Trust Africa Centre for Health and Population Studies, University of KwaZulu-Natal, Mtubatuba, South Africa; 5 School of Public Health, University of the Western Cape, Cape Town, South Africa

**Keywords:** Maternal health, Socio-economic inequalities, Access, Maternal delivery services

## Abstract

**Background:**

There is global concern with geographical and socio-economic inequalities in access to and use of maternal delivery services. Little is known, however, on how local-level socio-economic inequalities are related to the uptake of needed maternal health care. We conducted a study of relative socio-economic inequalities in use of hospital-based maternal delivery services within two rural sub-districts of South Africa.

**Methods:**

We used both population-based surveillance and facility-based clinical record data to examine differences in the relative distribution of socio-economic status (SES), using a household assets index to measure wealth, among those needing maternal delivery services and those using them in the Bushbuckridge sub-district, Mpumalanga, and Hlabisa sub-district, Kwa-Zulu Natal. We compared the SES distributions in households with a birth in the previous year with the household SES distributions of representative samples of women who had delivered in hospitals in these two sub-districts.

**Results:**

In both sub-districts, women in the lowest SES quintile were significantly under-represented in the hospital user population, relative to need for delivery services (8% in user population vs 21% in population in need; p < 0.001 in each sub-district). Exit interviews provided additional evidence on potential barriers to access, in particular the affordability constraints associated with hospital delivery.

**Conclusions:**

The findings highlight the need for alternative strategies to make maternal delivery services accessible to the poorest women within overall poor communities and, in doing so, decrease socioeconomic inequalities in utilisation of maternal delivery services.

## Background

Internationally, there is increased recognition that high levels of intra-country disparities in maternal health outcomes are as important to tackle as international disparities if the 2015 Millennium Development Goal of a reduction in maternal mortality is to be achieved. The ongoing discussions on the goal for ending preventable maternal mortality and the post-2015 development agenda highlight the need for ensuring that care is provided universally but also to those who need it most. Using data from the South Africa Demographic and Health Surveys (DHS) and Multiple Indicator Cluster Surveys, *national level* inequalities in access to maternal health services have been documented along geographical (regional, urban–rural) socio-economic and, at times, ethnic or racial lines [[1],[2]]. At the national level, inequalities are most pronounced in access to skilled birth attendance and comprehensive emergency obstetric care (CEOC) [[3],[4]] and considerably wider than inequalities in access to antenatal care (ANC) and childhood immunisation [[5]].

South Africa's Maternal Mortality Ratio (MMR) at 310 per 100 000 live births is high for a middle-income country. While the devastating HIV/AIDS epidemic partly accounts for poor maternal outcomes, South Africa’s apartheid past and high levels of inequality are also seen to be contributing factors [[6]]. Although 2003 DHS data showed that on average 91% of women delivered with skilled attendance, there were significant urban–rural, racial and socio-economic inequalities in this parameter. In rural areas, skilled attendance at delivery was 85% compared to 94% in urban areas; delivery in a hospital was 67% in rural areas compared to 80% in urban areas [[7]]. Inequalities in socio-economic status (SES) have a similar profile: 65% of the women in the poorest quintile delivering in hospitals compared to 89% of women in the SES-highest quintile. Wabiri et al. [[8]] in an analysis of national household survey data, concluded that while inequalities in SES in many indicators of access to maternal healthcare were small, women in the poorest quartile attended ANC later compared to relatively wealthier women (though attendance itself was high), and had lower skilled attendance rates. While it is known that *nationally*, maternal health services are not equally utilised, what is not known is the degree of SES inequality in maternal care service utilization at the *district* or *sub*-*district* level.

There has been a strong commitment from national government to tackle maternal mortality. Initiatives have included prioritising pregnant women for antiretroviral (ARV) treatment, appointment of district maternal and child health (MCH) teams, training community health workers in MCH, and plans to increase the availability of waiting homes and obstetric ambulances. In many rural areas, women are encouraged to deliver in hospitals or in health centres close to hospitals where there is ready access to Caesarean section, blood transfusions and other elements of comprehensive emergency obstetric care.

In this study, we thus examine for the first time socio-economic inequalities in utilisation of maternal delivery in public-sector hospitals in two rural sub-districts of South Africa, where established Health and Demographic Surveillance System (HDSS) sites provide an opportunity for such a study. Initiatives to reduce maternal mortality will be strengthened by a better understanding of socioeconomic gradients in the use of maternal health care faced by poorer women in rural areas. The district/sub-district level is important as it is the most decentralised managerial level of the health system where an appropriate fit between health care need and access can be ensured.

This analysis of maternal delivery forms one part of a larger study (REACH: Researching Equity in ACcess to Healthcare) funded by the Canadian International Development Research Centre, which explores inequalities in access and use of health care in South Africa of three health interventions: maternal health deliveries, tuberculosis treatment and anti-retroviral therapy for HIV.

## Methods

We conducted an analysis of inequality in utilization of hospital-based maternal delivery services in the Bushbuckridge and Hlabisa health sub-districts of Mpumalanga and Kwa-Zulu Natal Provinces, respectively. These two sub-districts were chosen because they both have HDSS providing population-level data on SES, births, and location of delivery. In the analysis, the SES of households with a birth in a woman 18 years or older in the previous year, obtained from HDSS data, was compared with the household SES, obtained from a representative sample of women, 18 years or older, who had delivered in hospitals in the two sub-districts. In 2009, 90.4% and 79.4% of maternal deliveries in Bushbuckridge and Hlabisa, respectively, took place in the formal health system (i.e. with skilled attendance). Of these, the vast majority (95% in Bushbuckridge and 92% in Hlabisa) occurred in hospital facilities [[9]]; hence the decision to conduct interviews at hospitals. Pregnancies that terminated in abortions or where the outcome was unknown were not included in the analysis.

### Population level data

The Agincourt HDSS (AHDSS) consists of an annual census of approximately 107500 people (as of May 2013) in an area of Bushbuckridge [[10]]. The following data were extracted from the AHDSS for the year 2007 from the 10,511 households with complete socio-economic data: number of pregnancies and their outcomes, maternal age and education, household characteristics, namely type of material used to construct the house walls and roof, access to water, toilet type, fuel used to cook, and ownership of assets such as a TV, fridge, stove, radio, landline telephone, vehicle, bicycle, and livestock. The 1,527 households with a woman over 18 years of age who had delivered in the preceding year were defined as the households needing maternal health services. The Africa Centre Demographic Information System (ACDIS) collects similar data on approximately 85,000 people in an area of Hlabisa [[11]]. Data from this database were extracted for 2009 on 8,448 households with complete socio-economic data and the subset of 1,491 households with a woman over 18 years of age who had delivered in the preceding year. Additional data on ownership of the following assets were available from this census: bed-nets, bed, block-maker, car battery, hot plate, kettle, gas cooker, kombi (vehicle), sink, motorcycle, primus stove, sofa, sewing machine, table and chairs, DVD player and wheelbarrow. Household characteristics and assets from both datasets were used to estimate an SES measure for each household in the two populations.

### SES index

We use Multiple Correspondence Analysis (MCA) to create an SES index. MCA, an extension of Correspondence Analysis, is used to measure the relationships between several categorical variables. MCA aims to decrease high dimensional data space through finding dimensions that capture the largest amount of information common to all the variables [[12]]. The SES index was computed separately for each sub-district using the HDSS population data on access to basic services (water, electricity, sanitation), type of house, and the household assets listed above. We only use the index formed by the first dimension identified in MCA, as this index already captures a very large proportion of the common information between the socioeconomic variables (79% and 74% in Bushbuckridge and Hlabisa, respectively). Once the continuous SES index was constructed for each sub-district, households were ranked by SES and grouped into quintiles ranging from lowest to highest SES. We use this relative measure of socioeconomic status, SES quintiles, to allow comparison of SES gradients across the two sub-districts included in our analyses. The absolute values of the continuous indices cannot be directly compared because their meanings differ in the communities.

### Sub-district level user data

We conducted patient exit interview surveys in women over 18 years of age delivering in one of the three hospitals in the two sub-districts (two in Bushbuckridge and one in Hlabisa) during the study period. Based on a Chi-squared Goodness-of-Fit test, we estimated that a sample of 300 women per sub-district would be required to detect SES differences with 80% power. In Bushbuckridge the sample was distributed proportional to the number of deliveries in each of the two hospitals. Respondents were recruited systematically at the time of discharge from the post-natal ward until the required sample size was achieved in each facility. Trained interviewers carried out the exit interviews in the local language of the respondent, collecting socio-economic data, as well as additional access variables related to the geographic accessibility, financial affordability, and cultural acceptability of hospital delivery services. During the course of the survey, a structured quality inventory on health systems inputs, processes and outputs was also completed in each hospital to measure the hospital capacity for comprehensive emergency obstetric care.

Data were collected over a period of 15 months, from June 2008 through September 2009. Ethical clearance for this study was obtained from the Universities of Cape Town, Witwatersrand and KwaZulu Natal, and provincial and local Departments of Health authorized the study. Written, informed consent was obtained from each participant in the exit interviews.

### Comparing the SES distributions of people needing and people using maternal delivery services

The SES distribution of the population in each sub-district, categorised into quintiles, was used to compare the SES distributions of the women needing and using maternal delivery services. We determined first the proportion of women in each quintile in need of maternal delivery services and then the proportion of women in each quintile who actually used these services. In this way these SES distributions are directly comparable. In order to test for trends and associations between those needing maternal delivery services and those who used these services, the Partitions of Pearson’s Chi-squared test for ordered columns, a contingency table analysis of ordered categorical variables (such as quintiles), was conducted [[13]].

## Results

The maternal health and service profile of the two sub-districts is summarised in Table 1. While both sites are considered poor rural areas, they nevertheless have different degrees of absolute disadvantage. Hlabisa sub-district is a more remote rural area than Bushbuckridge, and households have less access to piped water, and higher HIV prevalence and MMR than in Bushbuckridge. All three hospitals surveyed for this study were able to perform the signal functions of comprehensive emergency obstetric care (parenteral antibiotics, oxytocic drugs and anticonvulsants, facilities for the manual removal of placenta and retained products, assisted vaginal delivery and caesarian sections, and blood transfusion) [[14]]. In addition, the regional hospital in Bushbuckridge had a specialist obstetrician able to deal with complex obstetric emergencies.

**Table 1:** **Socio**-**demographic**, **maternal health and service profile of study sub**-**districts**

	**Bushbuckridge**		**Hlabisa**	**Source**
Population (2007)	509,970		150,557	[[15]]
Household access to piped water (2008/9)	90.0%		58.0%	[[16]]
Percentage of births with skilled attendant (Whole district) (2008/9)	90.4%		79.4%	[[16]]
Percentage of skilled attendant births occurring in hospitals (2008/9)^1^	94.6%		91.0%	
Maternal mortality ratio (period)	335/100,000 (2000–5)		769/100,000 (2000–7)	[[17],[18]]
Antenatal HIV prevalence	34.9%		39.9%	[[16]]
Emergency Obstetric Care Signal Functions^2^				
	Regional	District	District	
Caesarian section rate (%)	12.0%	14.0%	19.0%	
Forceps or vacuum equipment (for assisted vaginal delivery)	Available	Available	Available	
Oxytocic drugs	Available	Available	Available	
Anticonvulsants	Available	Available	Available	
Blood transfusion	Available	Available	Available	

^1^Personal Communication. Candice Day. Using 2008/2009 DHIS data.

^2^Data collected in a quality of care inventory done during the research project, and routine data for three months collected at the time that the quality of care inventory was carried out.

The vast majority of facility births in the two sub-districts occurred in the hospitals (94.6% in Bushbuckridge and 91.0% in Hlabisa), with the balance of births at a facility taking place in community health centres and in local clinics.

**Figure 1:**
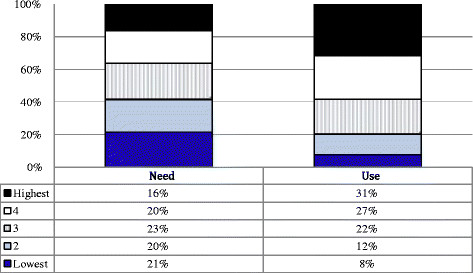
Utilisation of maternal delivery services analysis results – Bushbuckridge (Partitions of Pearson’s χ2 Test for for ordered columns: χ2 = 31.78 (p<0.001), Location: χ2 = 29.93 (p<0.001), Dispersion: χ2 = 0.7498 (p = 0.3866)).

**Figure 2:**
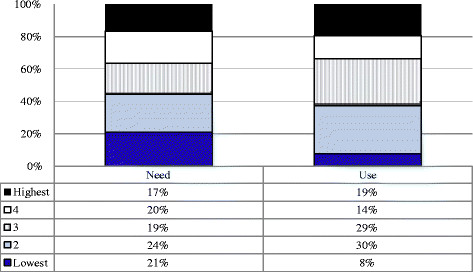
Utilisation of maternal delivery services analysis analysis results – Hlabisa (Partitions of Pearson’s χ2 Test for ordered columns: χ2 = 105.4 (p<0.001), Location: χ2 = 0.09764 (p = 0.7564), Dispersion: χ2 = 30.6 (p<0.001)).

Figures 1 and 2 present the findings of the utilisation analysis in the two sub-districts. The population SES distribution was decomposed into even quintiles ranked from lowest to highest, and compared with the SES of households needing maternal health services (‘Need’) and with the household SES of those delivering in hospitals (‘Use’). A total of 599 exit interviews – 299 in Bushbuckridge and 300 in Hlabisa – provided the data on the SES profile of the hospital user population.

In both sub-districts, the SES distribution of those needing maternal delivery services was not vastly different to that of the population, with the exception of a lower proportion of pregnancies among those women from households in the highest quintile. However, the SES distribution of the hospital user group showed distinct “pro-rich” patterns. In Bushbuckridge, users in the highest two quintiles comprised nearly 60% of all users. Further, while 41% of those needing maternal delivery services belonged to the lowest two quintiles, only 20% of users in our sample were from these two quintiles. The Partitions of Pearson’s Chi-squared test shows highly significant overall differences between need and use (p-value < 0.001) and further significant differences in location in the SES distribution between need and use (p-value < 0.001).

In Hlabisa, while use appeared to be relatively equitable among the highest 4 quintiles, women in the lowest quintile were under-represented in the user group (Figure 2). As with Bushbuckridge, women in this quintile comprised only 8% of hospital users, despite having 20% of the need. The Partitions of Pearson’s Chi-squared test also shows significant overall differences between need and use (p-value < 0.001) and further significant differences in dispersion across the SES distribution between need and use (p-value < 0.001).

Access to maternal delivery services was explored through three interlinked dimensions: availability (e.g. proximity of the health facility to the population served), affordability (e.g. the association between the health service related costs and the ability of households to meet these costs) and acceptability (e.g. expectations of and providers’ attitudes to patients and vice versa) [[19]]. Indicators of the access experience of women delivering in the hospitals, along these three dimensions are presented in Table 2. These are also reported in greater detail elsewhere [[20]]. Differences in categorical variables between the two study sites are tested using a Chi-squared test of association, while differences in continuous variables between study sites are compared using the Wilcoxon Rank sum test. As expected, the vast majority of women required transport to reach the hospital, with mean travel times increasing to nearly two hours (109 minutes) in Hlabisa. Women in Hlabisa appeared to have better access to ambulance services, while in Bushbuckridge, more than half (53%) had to hire a car to get to the hospital. When all health expenses (transport, supplies, food, and childcare) were taken into account, the costs of the delivery (despite absence of user fees) constituted on average, half the monthly household expenditure in the two sites. One in seven women reportedly had to borrow money or sell assets in order to meet the costs of the delivery. One in four women in Bushbuckridge and nearly one in two women in Hlabisa felt that health workers were too busy to listen to their problems; one in three felt that health workers did not respect them.

**Table 2:** Experiences of access amongst women delivering in hospital

	**Bushbuckridge**	**Hlabisa**	**Statistic**
	**(n = 299)**	**(n = 300)**	**p**-**value**
Availability			
Transport to facility:			χ^2^ = 143.600
Foot	12 (4.0%)	17 (5.8%)	p <0.000
Bicycle	1 (0.3%)	1 (0.3%)	
Taxi	109 (36.5%)	95 (32.5%)	
Bus	1 (0.3%)	7 (2.4%)	
Car	157 (52.5%)	47 (16.1%)	
Ambulance	19 (6.4%)	125 (42.8%)	
Mean time to hospital (minutes) (95% CI)	48.4 ( 43.8, 53.0)	109.6 (98.4, 120.9)	z = 10.997
			p < 0.000
Affordability			
Total costs of delivery as a percentage of household spending	51.4%	49.7%	z = −0.095
			p = 0.9244
Number of households borrowing or selling assets to pay for health care	40 (13.4%)	44 (14.7%)	χ^2^ = 0.211
			p = 0.6501
Acceptability			
Health worker too busy to listen to my problems	65 (25.6%)	137 (45.7%)	χ^2^ = 23.931
			p <0.000
Health worker respects me	188 (62.9%)	197 (65.9%)	χ^2^ = 0.593
			p = 0.4421

## Discussion

In two poor rural sub-districts in South Africa, those who are poor in relative terms compared to other members of the same community are most likely to belong to the small proportions of women who currently do not use hospital-based maternal delivery services. Inequalities in use of hospital based delivery services in these two rural sub-districts in South Africa confirm findings elsewhere [[3]] that access to delivery services is a particular problem for the poorest women within overall poor communities. We find that high proportions of women using hospital-based maternal delivery services needed to borrow money or sell assets to fund this health care service, suggesting that affordability is a major barrier to care. In a context of free maternal health care at the point of use, low affordability relates principally to the costs of travelling to a hospital facility, especially during labour.

The policy relevance of these findings are that strategies that seek to improve utilisation may at present not sufficiently lower the barriers of cost and distance to maternal care for women in the lowest SES quintile. The emphasis on delivery in hospitals where emergency obstetric care is potentially available, even if not needed, may present insurmountable barriers for poor women. Although not examined in this study, it is possible that the differentials in use also relate to non-financial barriers, such as less respect or attention being shown by providers to poorer women. We can further not rule out that alternative preferences and attitudes towards hospital-based deliveries among the poorest women in the two sub-districts contributed to the access inequalities observed in our study.

In theory, it is possible to manage normal vaginal deliveries at primary care clinics, which are more accessible to the poorest women within the sub-districts. However, this delivery would clearly involve ensuring that clinics were equipped and staffed to handle basic obstetric care – as well as an investment in obstetric ambulances and defined and agreed referral criteria. These conditions are currently not met in the two sub-districts. Cleary et al. [[21]] performed a similar study on antiretroviral treatment in two urban sites in South Africa where treatment was provided *at the clinic level*, finding no significant difference between the SES distribution of HIV positive individuals (as a proxy for those needing ARVs) and a random sample of users of antiretroviral treatment. This result and our findings jointly suggest that the location of health care and geographical barriers are critical determinants of access.

Our study is the first to document local-level inequalities in use of maternal delivery services, even within two of the overall poorest districts in South Africa. The District Health Barometer’s deprivation index, a measure of relative deprivation of populations across districts within South Africa, has placed Umkhanyakude District (containing Hlabisa) in the poorest 20% of districts and Ehlanzeni District (containing Bushbuckridge) in the second poorest 20% of districts in 2010/2011 [[9]]. However, the two districts display different patterns of inequality in access to hospital-based maternal delivery. This finding implies that policies to improve access among the poorest women in a district needs to be specific to the local context. For example, different patterns of transport use in the two districts studied, suggest that there is probably not a ’one size fits all’ policy solution to this problem for all districts.

## Conclusion

We find substantial socio-economic inequalities in use of hospital delivery services within two poor, rural districts in South Africa. Although the inequality patterns were different, in both sub-districts the women in the lowest quintile were least likely to use hospital-based delivery. Because the overwhelming majority of women in the sub-districts in this study do access hospital-based maternal delivery, these results suggest the need to shift the policy focus from ensuring overall access towards ensuring access particularly for the poorest women within the sub-districts.

## Abbreviations

DHS: Demographic and Health Surveys

CEOC: Comprehensive emergency obstetric care

ANC: Antenatal care

MMR: Maternal mortality ratio

SES: Socio-Economic status

ARV: AntiRetroViral

MCH: Maternal and child Health

HDSS: Health and Demographic Surveillance System

REACH: Researching Equity in ACcess to Healthcare

AHDSS: Agincourt HDSS

ACDIS: Africa centre demographic information system

MCA: Multiple correspondence analysis

## Competing interests

The authors declare that they have no competing interests.

## Authors’ contributions

HS, SPS and LPK conceptualized the paper. SPS managed and analysed the data. SPS and LPK wrote the manuscript. TB and HS reviewed and provided extensive comments on the analytical methods and the manuscript. All authors read and approved the manuscript.
